# Adverse childhood experiences and COVID-19 vaccination uptake: Examining the intersection of sex and urban-rural residence

**DOI:** 10.1371/journal.pone.0336390

**Published:** 2025-11-07

**Authors:** Karyn Fu, Dylan B. Jackson, Alexander Testa

**Affiliations:** 1 Rice University, Houston, Texas, United States of America; 2 Johns Hopkins Bloomberg School of Public Health, Baltimore, Maryland, United States of America; 3 Department of Management, Policy and Community Health, School of Public Health, University of Texas Health Science Center at Houston, Houston, Texas, United States of America; Medical College of Wisconsin, UNITED STATES OF AMERICA

## Abstract

**Background:**

Adverse childhood experiences (ACEs) have been linked to negative health outcomes and behaviors in adulthood. Despite widespread research on ACEs, their relationship with COVID-19 vaccination uptake, particularly heterogeneity across demographic groups, remains underexplored. This study examined the association between ACEs and COVID-19 vaccination status, with a focus on how this relationship varies by sex and urban-rural residence.

**Methods:**

Data were obtained from the 2022 Behavioral Risk Factor Surveillance System (BRFSS), including respondents who participated in the ACEs and COVID-19 vaccination optional state modules (N = 12,085 adults). COVID-19 vaccination status (yes/no) served as the dependent variable, while ACEs were categorized into four levels: 0, 1, 2–3, and 4 + ACEs. Multivariable logistic regression analyses, stratified by sex and urban-rural residence, were conducted to assess the association between ACEs and vaccination status.

**Results:**

Among the sample, 76.2% reported receiving at least one dose of the COVID-19 vaccine. Multivariable analysis revealed no statistically significant association between ACEs and vaccination status for the full sample. Stratified analyses indicated that male respondents living in rural counties with 4 + ACEs had significantly lower odds of vaccination (adjusted odds ratio [aOR] = 0.57, 95% CI = 0.34–0.96). No significant associations were observed for other demographic groups (e.g., females in urban or rural areas; males in urban areas).

**Conclusions:**

The findings suggest that males in rural areas with high ACE exposure may be vulnerable to low COVID-19 vaccination uptake. Targeted trauma-informed public health interventions warrant consideration to address vaccination uptake among this population.

## Introduction

The COVID-19 pandemic (2020−2023) was the deadliest disease outbreak in United States history [1], leading to over 103.4 million disease cases and 1.1 million deaths as of July 2024 [2]. Beyond the devastating toll to human life, the COVID-19 pandemic generated immense economic losses, estimated to be as great as $14 trillion by the end of 2023 [3]. The profound, widespread consequences of the COVID-19 pandemic quickly spurred efforts for vaccine development. Once vaccines were made available to Americans ages 16 and older in December 2020, they became a central element of the United States pandemic response plan [4]. Within the first six months of implementation, the COVID-19 vaccine averted an estimated eight million cases and 120,000 deaths [4]. Numerous studies have also consistently demonstrated COVID-19 vaccination is associated with lower COVID-19 incidence and mortality rates [5–8].

Despite the public health benefits of the COVID-19 vaccine, the United States’ efforts to increase vaccination have been characterized by high levels of vaccine hesitancy [9]—defined as the delay in acceptance or refusal of vaccination despite the availability of vaccination services [10]. Indeed, one recent systematic review of COVID-19 vaccine hesitancy in the United States estimated 15.6% to 46.4% of the population espoused hesitancy toward COVID-19 vaccination [11]. These statistics were then mirrored in vaccine uptake trends. For example, as of June 2024, 19.0% of American adults have never received a COVID-19 vaccine [9]. Lack of vaccination was also elevated in certain demographic populations, including males (21.3% compared to 16.6% for females) and those living in rural areas (29.9% compared to 15.8% in urban areas; 18.2% in suburban areas) [9]. Even so, there is a dearth of research investigating how prominent life experiences, such as early life adversity, influence COVID-19 vaccine uptake and how these relationships may vary across demographic correlates of COVID-19 vaccination.

Adverse childhood experiences (ACEs) are potentially traumatic events, including abuse, neglect, and household dysfunction, that occur before age 18 [12,13]. ACEs have been extensively characterized as a determinant of negative health outcomes and behaviors in adulthood, with particularly severe impacts observed for individuals with multiple ACEs exposures [13–16]. This, in part, is explained by individuals with a history of ACEs being more likely to exhibit health-risk behaviors [17,18] and having a lower uptake of preventive services [19–21].

The health sequelae of ACEs are especially relevant in the context of COVID-19 vaccine uptake. The psychological and socioeconomic impacts of ACEs may amplify barriers to vaccination, such as cost and accessibility [22–24], and erode trust in medical institutions [25–27]—factors associated with low COVID-19 vaccine uptake in the general population [28]. Nevertheless, the relationship between ACEs and COVID-19 vaccination status has only begun to be explored. Limited research to date has demonstrated that individuals with a greater number of ACEs are more likely to report COVID-19 vaccine hesitancy [25,29] and less likely to accept a COVID-19 vaccine [30], compared to those with no ACEs.

Beyond the direct effects of ACEs on COVID-19 vaccine uptake, there is also a strong rationale to expect this relationship to vary across segments of the population. To be sure, there are well-documented disparities in both ACE exposure [14,31–33] and COVID-19 vaccine uptake by sex and geography [9], with males and those living in rural areas having a greater probability of being exposed to ACEs and a lesser probability of receiving a COVID-19 vaccine [31–33]. Thus, there is reason to believe the relationship between ACEs and COVID-19 vaccination will also vary by sex and urban/rural residence, and potentially the intersection of sex and geographic residence, yet such a possibility has not been previously investigated by extant research. Therefore, understanding how these intersecting identities and environments influence vaccine hesitancy can reveal unique barriers faced by specific populations, guiding tailored public health interventions to improve vaccine uptake.

Using a national sample of Americans from the 2022 Behavioral Risk Factor Surveillance System (BRFSS), the current study aimed to fill gaps in knowledge regarding the relationship between ACEs and COVID-19 vaccination by addressing the following research questions:

1. What is the relationship between ACEs and COVID-19 vaccination status in adulthood?
2. Does the relationship between ACEs and COVID-19 vaccination status in adulthood vary by the intersection of sex and urban/rural residence?

## Materials and methods

### Data

Data were from the 2022 Behavioral Risk Factor Surveillance System (BRFSS), an annual national cross-sectional telephone survey conducted in all 50 states, the District of Columbia, and three US territories. With technical and methodological assistance from the Centers for Disease Control and Prevention, state health departments used in-house interviewers or contracted with telephone call centers or universities to administer the BRFSS surveys continuously throughout the year. The states used a standardized core questionnaire, optional modules, and state-added questions. The survey was conducted using Random Digit Dialing (RDD) techniques on both landlines and cell phones in English or Spanish, to create a representative sample of the noninstitutionalized, civilian, adult (≥18 years) US population. In 2022, the BRFSS had a 45% response rate, completing 445,132 interviews in total [34].

The analytic sample for the current study is 12,085 respondents from three states—Arkansas, Iowa, and North Dakota—who participated in the optional state modules on “Adverse Childhood Experiences” and “COVID Vaccination” (refer to Appendix A for details of the sample selection procedures). The BRFSS survey was approved by the National Center for Health Statistics Research Ethics Review Board [35]. Because the BRFSS are de-identified and publicly available data, the use of the BRFSS does not qualify as human subjects research and was exempt from local institutional review board review for the purposes of this study.

### Dependent variable

*COVID-19 vaccination* was based on a yes or no question from the state optional module on “COVID vaccination,” asking, “Have you received at least one dose of a COVID-19 vaccination?” (refer to Appendix C for details) [30].

### Independent variable

*Adverse childhood experiences* was based on a series of questions from the state optional module “Adverse Childhood Experiences,” where respondents answered yes or no to retrospective questions on ACEs occurring before age 18. Included ACEs are (a) household mental illness, (b) household alcoholism, (c) household illegal drug use, (d) household incarceration, (e) parents divorced or separated, (f) household domestic violence, (g) physical abuse, (h) verbal abuse, and (i) sexual abuse (refer to Appendix B for details). Responses to these items were summed into a single scale and categorized into four levels of exposure: 0 ACEs, 1 ACE, 2–3 ACEs, or 4 or more ACEs [36–38].

### Moderating variables

Biological *sex* (male or female) was self-reported by the respondent. *Urban-rural classification* was a calculated variable characterizing residence in an urban or rural county based on the National Center for Health Statistics (NCHS) urban-rural classification scheme for counties. NCHS-categorized large central metropolitan counties, large fringe metropolitan counties (suburban areas), medium metropolitan counties, small metropolitan counties, and micropolitan counties were defined as urban counties in the BRFSS. NCHS-categorized non-core countries were defined as rural counties [39].

### Control variables

Control variables included respondent *age* (18–24, 25–34, 35–44, 45–54, 55–65, or 65+), *race/ethnicity* (non-Hispanic White, non-Hispanic Black, Hispanic, or non-Hispanic other race), *marital status* (married, divorced/separated, widowed, never married, or member of an unmarried couple), *educational attainment* (less than high school, high school graduate, some college, or college graduate), *child in home* (yes or no), *military veteran* (yes or no), *household income* ($ < 25,000, $25,000 - $49,999, $50,000 - $74,999, $75,000 - $99,999, $100,000 - $149,999 or ≥ $150,000), and *survey language* (English or Spanish).

### Analytic approach

Unweighted frequencies and weighted percentages were calculated. Multivariable logistic regression was used to assess the relationship between an individual’s number of ACEs and their COVID-19 vaccination status, adjusting for covariates. To investigate the moderation of this relationship by biological sex and urban/rural county residence, analyses were then stratified by the intersection of sex and urban-rural classification to create four separate multivariable regression models for the relationship between ACEs number and COVID-19 vaccination status: urban county and female, urban county and male, rural county and female, rural county and male. Analyses were adjusted for BRFSS survey weights using the *svy* command in STATA v.18.5 (StataCorp). Statistical significance was determined at the *p* < .05 threshold (two-tailed).

## Results

Among the 12,085 adults in the sample, 76.2% reported receiving at least one dose of the COVID-19 vaccine. The greatest proportion of respondents reported having 0 ACE exposure (34.1%), 22.0% reported 1 ACE, 22.5% reported 2–3 ACEs, and 21.4% reported 4 + ACEs. COVID-19 vaccination was highest among those with 0 ACEs (80.5%). The percentage of respondents reporting having received the COVID-19 vaccine decreased to 77.3% for those with 1 ACE, 76.5% for 2–3 ACEs, and 67.9% for 4 + ACEs (see Table 1). Results of a Chi-squared test revealed that the bivariate relationship between ACEs and COVID-19 status is statistically significant (*p *< .001).

**Table 1:** Weighted Summary Statistics of the Analytic Sample from the Behavioral Risk Factor Surveillance System, 2022 (N = 12,085).

	Full Sample (*N = *12,085)	0 ACEs (N = 4,791)	1 ACE (N = 2,736)	2-3 ACEs (N = 2,570)	4 + ACEs (N = 1,988)	*p*-value^a^
**Variable**						
COVID-19 Vaccination						<0.001
No	23.8% (2,421)	19.5% (807)	22.7% (537)	23.5% (510)	32.1% (567)	
Yes	76.2% (9,664)	80.5% (3,984)	77.3% (2,199)	76.5% (2,060)	67.9% (1,421)	
*Number of ACEs*						NA
0 ACEs	34.1% (4,791)	NA	NA	NA	NA	
1 ACE	22.0% (2,736)	NA	NA	NA	NA	
2-3 ACEs	22.5% (2,570)	NA	NA	NA	NA	
4 + ACEs	21.4% (1,988)	NA	NA	NA	NA	
*Age*						<.001
18-24	10.2% (700)	7.0% (180)	10.0% (160)	10.8% (170)	14.8% (190)	
25-34	17.1% (1,244)	12.3% (346)	14.2% (247)	19.7% (316)	25.1% (335)	
35-44	16.8% (1,617)	13.6% (485)	17.2% (363)	17.4% (378)	20.9% (391)	
45-54	14.8% (1,680)	13.0% (547)	14.9% (369)	15.7% (396)	16.9% (368)	
55-64	17.2% (2,293)	19.3% (898)	17.4% (518)	16.6% (494)	14.1% (383)	
65+	23.8% (4,551)	34.8% (2,335)	26.2% (1,079)	19.7% (816)	8.2% (321)	
*Sex*						<.001
Female	49.8% (6,086)	46.9% (2,320)	45.5% (1,295)	49.4% (1,305)	59.0% (1,166)	
Male	50.2% (5,999)	53.1% (2,471)	54.5% (1,441)	50.6% (1,265)	41.0% (822)	
*Race/Ethnicity*						<.001
Hispanic	5.4% (620)	4.4% (198)	5.3% (133)	6.4% (144)	6.1% (145)	
Non-Hispanic Black	7.0% (499)	6.2% (171)	7.6% (121)	7.5% (117)	7.2% (90)	
Non-Hispanic White	81.7% (10,514)	85.1% (4,304)	83.4% (2,408)	80.2% (2,199)	76.3% (1,603)	
Non-Hispanic Other	5.8% (452)	4.3% (118)	3.7% (74)	5.9% (110)	10.3% (150)	
*Marital Status*						<.001
Married	56.1% (6,720)	62.8% (2,878)	58.7% (1,571)	52.3% (1,344)	46.8% (927)	
Divorced or Separated	12.9% (1,639)	9.5% (491)	12.6% (358)	13.5% (392)	17.9% (398)	
Widowed	7.1% (1,403)	8.9% (706)	7.6% (325)	6.9% (267)	3.7% (105)	
Never Married	19.5% (1,867)	16.0% (597)	17.9% (401)	21.8% (449)	24.3% (420)	
Member of an Unmarried Couple	4.4% (456)	2.8% (119)	3.1% (81)	5.5% (118)	7.2% (138)	
*Educational Attainment*						<.001
Less than High School	7.6% (647)	6.0% (234)	7.0% (131)	7.9% (143)	10.3% (139)	
High School Graduate	30.3% (3,430)	28.0% (1,266)	32.6% (815)	29.6% (731)	32.2% (618)	
Some College	34.5% (3,518)	33.1% (1,342)	32.1% (756)	34.1% (743)	39.7% (677)	
College Graduate	27.7% (4,490)	33.0% (1,949)	28.3% (1 034 )	28.4% (953)	17.9% (554)	
*Child in Home*						<.001
No	65.6% (8,985)	72.6% (3,838)	66.0% (2 040 )	65.3% (1,873)	54.2% (1,234)	
Yes	34.4% (3,100)	27.4% (953)	34.0% (696)	34.7% (697)	45.8% (754)	
*Veteran Status*						0.105
No	88.9% (10,592)	89.1% (4,201)	88.5% (2,381)	87.7% (2,234)	90.5% (1,776)	
Yes	11.1% (1,493)	10.9% (590)	11.5% (355)	12.3% (336)	9.5% (212)	
*Household Income*						<.001
Less than $25,000	15.7% (1,868)	12.8% (649)	13.2% (365)	15.9% (420)	22.6% (434)	
$25,000 - $49,999	27.2% (3,299)	25.9% (1,269)	26.1% (753)	26.8% (700)	30.7% (577)	
$50,000 - $74,999	19.1% (2,332)	19.3% (966)	20.1% (552)	19.5% (459)	17.5% (355)	
$75,000 - $99,999	14.2% (1,778)	15.4% (729)	14.5% (417)	13.3% (367)	13.2% (265)	
$100,000 - $149,999	13.4% (1,603)	14.8% (671)	15.0% (364)	13.6% (352)	9.4% (216)	
$150,000 or more	10.3% (1,205)	11.8% (507)	11.1% (285)	10.8% (272)	6.5% (141)	
*Survey Language*						0.004
English	97.8% (11,761)	97.6% (4,656)	97.5% (2,657)	97.3% (2,494)	99.2% (1,954)	
Spanish	2.2% (324)	2.4% (135)	2.5% (79)	2.7% (76)	0.8% (34)	
*Urbanicity*						
Urban	75.7% (8,665)	72.4% (3,300)	74.3% (1,924)	78.5% (1,904)	79.5% (1,537)	
Rural	24.3% (3,420)	27.6% (1,491)	25.7% (812)	21.5% (666)	20.5% (451)	

^a^p-value represents the results from Chi-Squared tests. The table presents weighted percentages and unweighted frequencies in parentheses.

*Abbreviations:* NA = not applicable.

Table 2 reports the results of multivariable logistic regression analysis with reference categories set to respondents with no COVID-19 vaccination and 0 ACEs. The results showed that no statistically significant relationship is found between ACEs and COVID-19 vaccination among the full sample when accounting for control variables. In the sample, 36.1% of respondents were females living in urban counties, 35.6% were males living in urban counties, 14.2% were females living in rural counties, and 14.1% were males living in rural counties. When stratifying analyses by biological sex and urban/rural residence, the results demonstrated male respondents living in rural counties who experienced 4+ ACEs have 43% lower odds of COVID-19 vaccination than male respondents living in rural counties who experienced 0 ACEs (adjusted odds ratio [aOR] = 0.57, 95% Confidence Interval [CI] = 0.34–0.96). In contrast, no other sex and urban-rural classification categories revealed statistically significant associations between any of the ACE number categories and COVID-19 vaccination. Thus, the results indicated that the relationship between ACEs and COVID-19 vaccination status was concentrated among males in rural areas with high (4+) ACE exposure.

**Table 2:** Multivariable Logistic Regression of COVID-19 Vaccination on ACEs.

	Full Sample (N = 12,085)	Urban Female (N = 4,368)	Urban Male (N = 4,297)	Rural Female (N = 1,718)	Rural Male (N = 1,702)
**Number of ACEs** ^**a**^	**aOR (95% CI)**	**aOR (95% CI)**	**aOR (95% CI)**	**aOR (95% CI)**	**aOR (95% CI)**
0 (Reference)	NA	NA	NA	NA	NA
1	1.01 (0.85-1.20)	1.08 (0.76-1.53)	1.01 (0.76-1.34)	1.15 (0.72-1.85)	0.84 (0.59-1.21)
2-3	1.03 (0.86-1.23)	1.07 (0.77-1.48)	1.03 (0.78-1.37)	1.24 (0.78-1.95)	0.94 (0.61-1.45)
4+	0.91 (0.76-1.10)	0.87 (0.63-1.19)	1.08 (0.80-1.46)	1.07 (0.68-1.70)	0.57* (0.34-0.96)

*** p < 0.001, ** p < 0.01, * p < 0.05.

*Abbreviations*: ACEs = adverse childhood experiences; aOR = adjusted odds ratio; CI = confidence interval.

^a^Control variables include age, race/ethnicity, marital status, educational attainment, child in the home, veteran status, household income, and survey language. In the regression model of the full sample, sex and urbanicity act as additional control variables.

### Supplementary analyses

Appendix D reports separate multivariable logistic regression models examining the relationship between each type of ACE and COVID-19 vaccination. For the full sample, the results illustrated statistically significant associations between household illegal drug use (aOR = 0.82, CI = 0.68–1.00), household incarceration (aOR = 0.74, CI = 0.59–0.92), and sexual abuse (aOR = 0.70, CI = 0.58–0.85), and COVID-19 vaccination. For female respondents living in urban counties, household incarceration (aOR = 0.66, CI = 0.45–0.97) and sexual abuse (aOR = 0.66, CI = 0.50–0.88) remained significantly associated with COVID-19 vaccination. For male respondents living in rural counties, only household mental illness (aOR = 0.56, CI = 0.36–0.88) had a statistically significant relationship with COVID-19 vaccination. No ACE types demonstrated an association with COVID-19 vaccination for urban males and rural females.

## Discussion

The current study investigated the relationship between ACEs and COVID-19 vaccination status and how the relationship varied across biological sex and residence in an urban or rural area. The study findings revealed several notable patterns. First, while the relationship found a bivariate association between greater ACE exposure and COVID-19 vaccination, this relationship in the full sample was rendered statistically non-significant after controlling for potential confounding variables. However, upon further assessment of this relationship, stratified by intersections of sex and residence in an urban or rural area, the findings illustrated that males living in rural counties with 4+ ACE exposures were significantly less likely to receive the COVID-19 vaccination.

These results are somewhat consistent with previous literature. Prior work has found an association between a high number of ACEs and low COVID-19 vaccination or high COVID-19 vaccine hesitancy [25,30]. While we found a similar pattern in a bivariate association, we found no significant association between ACE exposure and COVID-19 vaccination status in the multivariable analyses. The current study is the first to the author’s knowledge to assess how the relationship between ACEs and COVID-19 vaccination status varies across key geographic and demographic characteristics, revealing that the relationship is concentrated among males living in rural counties with 4+ ACE exposures.

These findings demonstrated rural males with high childhood adversity should be considered a group of focus for future COVID-19 vaccination efforts. This is especially important because male sex and residence in a rural area are associated with severe COVID-19 illness [40–42]. ACEs are also related to several health outcomes and behaviors (i.e., heart disease, respiratory disease, tobacco use, poor diet) that are risk factors for severe COVID-19 illness [14]. Therefore, the low rates of COVID-19 vaccination uptake among this population may further contribute to rural males with high ACE exposure being at an elevated risk of severe cases of COVID-19.

Several past vaccination campaigns—including those put forth by the White House during the Biden-Harris administration—had included provisions for resources targeting the groups most susceptible to COVID-19 infection and/or serious complications from infection [43,44]. Rural males with high childhood adversity were never previously identified as a group for targeted COVID-19 vaccine messaging. While these past efforts have had interventions for rural communities, the failure to use targeted health communication messaging to increase vaccination among males with high ACE counts may mean these interventions were too broad to effect behavioral change in this specific population.

In terms of practical implications, it is also important to note that while this study uses a count of total ACE exposure, the ACE score was designed for population-level epidemiological research rather than individual-level practice [45]. Accordingly, while ACE scores can forecast mean group differences in health problems at the individual level, ACE scores have poor accuracy in identifying specific individuals at high risk for health problems [46]. Thus, while the results point to a specific segment of the population—rural males with high ACE exposure—as a group with lower uptake of COVID-19 vaccines and potentially greater vaccine hesitancy, future work is needed to understand whether and how targeted health communication strategies and vaccine campaigns may effectively integrate culturally tailored messaging [47] and trauma-responsive strategies to improve vaccine uptake among this segment of the population [ 48,49].

### Limitations and future directions

This study has limitations that can be expanded upon in future research. First, due to the self-report nature of the BRFSS, responses to ACEs and COVID-19 vaccination may have been affected by social desirability or recall bias. Second, due to small cell sizes across the intersection of race/ethnicity and urban/rural status, this study was underpowered to explore how COVID-19 vaccine status varied by racial-urban/rural intersections. A valuable direction for future research is to further assess the relationship between ACEs and COVID-19 vaccination status across multiple, intersectional identities. Third, the results are also limited to the COVID-19 vaccine and should not necessarily generalize to other vaccinations, like those for influenza or human papillomavirus. The cross-sectional nature of the BRFSS also prevents the drawing of causal inferences from the data. Future research using longitudinal data as well as mixed methods studies is important for understanding the possible mechanisms underlying the relationship in the current study. Furthermore, only three states included in the BRFSS elected to ask both the ACEs and COVID-19 vaccination questions, limiting the findings’ generalizability to the broader US population. Importantly, these three states (Arkansas, Iowa, and North Dakota) represent states that are more rural and politically conservative, on average, relative to the rest of the US. Thus, future research should seek to replicate the findings with a broader and more representative sample of the US population. Finally, the study uses data from the 2022 BRFSS, conducted after the peak of the COVID-19 pandemic waned. Therefore, our findings may not speak to COVID-19 vaccine hesitancy at earlier stages of the pandemic.

## Conclusions

The present study is the first to find that the relationship between high ACE exposure and COVID-19 vaccination is concentrated in males living in rural areas. Therefore, these findings demonstrate an important need for future work to consider how vaccination education and accessibility campaigns can be tailored to this demographic group. Future research should also aim to deepen our understanding of why rural males with more ACEs are less likely to have had the COVID-19 vaccine. Such efforts are paramount to further improving vaccination interventions and pandemic preparedness and reducing the societal burden of COVID-19.

## Supporting information

S1 File Appendices A to D. (DOCX)

## References

[1] 1918 pandemic (H1N1 virus) | pandemic influenza (flu) | CDC. https://archive.cdc.gov/www_cdc_gov/flu/pandemic-resources/1918-pandemic-h1n1.html. 2023. Accessed 2024 August 8.

[2] COVID-19 cases | WHO COVID-19 dashboard. https://data.who.int/dashboards/covid19/cases. Accessed 2024 July 29.

[3] Walmsley T, Rose A, John R, Wei D, Hlávka JP, Machado J. Macroeconomic consequences of the COVID-19 pandemic. Econ Model. 2023;120:106147. 36570545 10.1016/j.econmod.2022.106147 PMC9768433

[4] Yamana TK, Galanti M, Pei S, Di Fusco M, Angulo FJ, Moran MM, et al. The impact of COVID-19 vaccination in the US: Averted burden of SARS-COV-2-related cases, hospitalizations and deaths. PLoS One. 2023;18(4):e0275699. doi: 10.1371/journal.pone.0275699 37098043 PMC10129007

[5] Suthar AB, Wang J, Seffren V, Wiegand RE, Griffing S, Zell E. Public health impact of covid-19 vaccines in the US: observational study. BMJ. 2022;377:e069317. doi: 10.1136/bmj.n317 PMC9044401 35477670

[6] Dye C. The benefits of large scale covid-19 vaccination. BMJ. 2022;377:o867. 10.1136/bmj.o867 35477535

[7] Hoxha I, Agahi R, Bimbashi A, Aliu M, Raka L, Bajraktari I. Higher COVID-19 vaccination rates are associated with lower COVID-19 mortality: a global analysis. Vaccines. 2023;11(1):74. 10.3390/vaccines11010074 PMC9862920 36679919

[8] Huang Y-Z, Kuan C-C. Vaccination to reduce severe COVID-19 and mortality in COVID-19 patients: a systematic review and meta-analysis. Eur Rev Med Pharmacol Sci. 2022;26(5):1770–6. doi: 10.26355/eurrev_202203_28248 35302230

[9] COVID-19 Vaccination Coverage and Vaccine Confidence Among Adults. CDC. https://www.cdc.gov/vaccines/imz-managers/coverage/covidvaxview/interactive/adults.html. 2024. Accessed 2024 July 29.

[10] MacDonald NE. Vaccine hesitancy: definition, scope and determinants. Vaccine. 2015;33(34):4161–4. doi: 10.1016/j.vaccine.2015.04.036 25896383

[11] Wang Y, Liu Y. Multilevel determinants of COVID-19 vaccination hesitancy in the United States: A rapid systematic review. Prev Med Rep. 2021;25:101673. doi: 10.1016/j.pmedr.2021.101673 34934611 PMC8675390

[12] CDC. About Adverse Childhood Experiences. https://www.cdc.gov/aces/about/index.html. 2024. Accessed 2024 July 29.

[13] Felitti VJ, Anda RF, Nordenberg D, Williamson DF, Spitz AM, Edwards V, et al. Relationship of childhood abuse and household dysfunction to many of the leading causes of death in adults. The Adverse Childhood Experiences (ACE) Study. Am J Prev Med. 1998;14(4):245–58. doi: 10.1016/s0749-3797(98)00017-8 9635069

[14] Petruccelli K, Davis J, Berman T. Adverse childhood experiences and associated health outcomes: A systematic review and meta-analysis. Child Abuse Negl. 2019;97:104127. doi: 10.1016/j.chiabu.2019.104127 31454589

[15] Bellis MA, Hughes K, Ford K, Rodriguez GR, Sethi D, Passmore J. Life course health consequences and associated annual costs of adverse childhood experiences across Europe and North America: a systematic review and meta-analysis. Lancet Public Health. 2019;4(10):e517-28. 10.1016/S2468-2667(19)30145-8 PMC7098477 31492648

[16] Hughes K, Bellis MA, Hardcastle KA, Sethi D, Butchart A, Mikton C, et al. The effect of multiple adverse childhood experiences on health: a systematic review and meta-analysis. Lancet Public Health. 2017;2(8):e356–66. doi: 10.1016/S2468-2667(17)30118-4 29253477

[17] Campbell JA, Walker RJ, Egede LE. Associations Between Adverse Childhood Experiences, High-Risk Behaviors, and Morbidity in Adulthood. Am J Prev Med. 2016;50(3):344–52. doi: 10.1016/j.amepre.2015.07.022 26474668 PMC4762720

[18] Espeleta HC, Brett EI, Ridings LE, Leavens ELS, Mullins LL. Childhood adversity and adult health-risk behaviors: Examining the roles of emotion dysregulation and urgency. Child Abuse Negl. 2018;82:92–101. doi: 10.1016/j.chiabu.2018.05.027 29879586

[19] Alcalá HE, Valdez-Dadia A, von Ehrenstein OS. Adverse childhood experiences and access and utilization of health care. J Public Health (Oxf). 2018;40(4):684–92. doi: 10.1093/pubmed/fdx155 29182751 PMC6306082

[20] Miller-Cribbs JE, Wen F, Coon KA, Jelley MJ, Foulks-Rodriguez K, Stearns J. Adverse childhood experiences and inequities in adult health care access. Int Public Health J. 2016;8(2):257–70.

[21] Testa A, Jackson DB, Vaughn MG, Ganson KT, Nagata JM. Adverse Childhood Experiences, health insurance status, and health care utilization in middle adulthood. Soc Sci Med. 2022;314:115194. doi: 10.1016/j.socscimed.2022.115194 36283330

[22] Currie J, Widom CS. Long-term consequences of child abuse and neglect on adult economic well-being. Child Maltreat. 2010;15(2):111–20. doi: 10.1177/1077559509355316 20425881 PMC3571659

[23] Zielinski DS. Child maltreatment and adult socioeconomic well-being. Child Abuse Negl. 2009;33(10):666–78. doi: 10.1016/j.chiabu.2009.09.001 19811826

[24] Zhang S, Richardson BA, Lin J, Winer RL. The Association Between Adverse Childhood Experiences and Human Papillomavirus Vaccination Coverage in US Young Adults: A Cross-Sectional Study. Sex Transm Dis. 2023;50(10):656–63. 37432983 10.1097/OLQ.0000000000001846

[25] Bellis MA, Hughes K, Ford K, Madden HCE, Glendinning F, Wood S. Associations between adverse childhood experiences, attitudes towards COVID-19 restrictions and vaccine hesitancy: a cross-sectional study. BMJ Open. 2022;12(2):e053915. doi: 10.1136/bmjopen-2021-053915 35105582 PMC8829847

[26] Munoz RT, Hanks H, Brahm NC, Miller CR, McLeod D, Fox MD. Adverse Childhood Experiences and Trust in the Medical Profession among Young Adults. J Health Care Poor Underserved. 2019;30(1):238–48. doi: 10.1353/hpu.2019.0018 30827980

[27] Bellis MA, Hughes K, Ford K, Sharp C, Hill R. Associations between adverse childhood experiences and trust in health and other information from public services, professionals and wider sources: national cross sectional survey. BMJ Public Health. 2024;2(1):e000868. doi: 10.1136/bmjph-2023-000868 40018105 PMC11812900

[28] Troiano G, Nardi A. Vaccine hesitancy in the era of COVID-19. Public Health. 2021;194:245–51. 33965796 10.1016/j.puhe.2021.02.025 PMC7931735

[29] Moffitt TE, Caspi A, Ambler A, Bourassa K, Harrington H, Hogan S, et al. Deep-seated psychological histories of COVID-19 vaccine hesitance and resistance. PNAS Nexus. 2022;1(2):pgac034. doi: 10.1093/pnasnexus/pgac034 35783503 PMC9245853

[30] Chau-Valdivia CA. Examining the relationship between adverse childhood experiences and COVID-19 vaccine status using 2022 BRFSS data: A cross-sectional study. J Public Health. 2023.

[31] Solberg MA, Blair LM, Schlegel EC, Kurzer JAMJ. Health disparities among sexual and gender minorities with adverse childhood experiences: insights from the 2021 behavioral risk factor surveillance system data. Am J Public Health. 2023;113(12):1343–51. doi: 10.2105/AJPH.2023.307420 37939340 PMC10632852

[32] Dosanjh LH, Hinds JT, Cubbin C. The impacts of adverse childhood experiences on socioeconomic disadvantage by sexual and gender identity in the U.S. Child Abuse Negl. 2023;141:106227. 37163969 10.1016/j.chiabu.2023.106227

[33] Crouch E, Radcliff E, Probst JC, Bennett KJ, McKinney SH. Rural-Urban Differences in Adverse Childhood Experiences Across a National Sample of Children. J Rural Health. 2020;36(1):55–64. doi: 10.1111/jrh.12366 30938864

[34] Centers for Disease Control and Prevention. Behavioral Risk Factor Surveillance System 2022 Summary Data Quality Report. 2023.

[35] Behavioral Risk Factor Surveillance System. https://www.cdc.gov/brfss/index.html. Accessed 2024 November 27.

[36] Swedo EA, Aslam MV, Dahlberg LL, Niolon PH, Guinn AS, Simon TR, et al. Prevalence of Adverse Childhood Experiences Among U.S. Adults — Behavioral Risk Factor Surveillance System, 2011–2020. MMWR Morb Mortal Wkly Rep. 2023 Jun 30;72(26):707–15. 37384554 10.15585/mmwr.mm7226a2 PMC10328489

[37] Merrick MT, Ford DC, Ports KA, Guinn AS, Chen J, Klevens J. Vital Signs: Estimated Proportion of Adult Health Problems Attributable to Adverse Childhood Experiences and Implications for Prevention — 25 States, 2015–2017. MMWR Morb Mortal Wkly Rep. 2019;68(44):999–1005. 31697656 10.15585/mmwr.mm6844e1 PMC6837472

[38] Testa A, Fu K, Jackson DB, Semenza DC, McKay S. Adverse Childhood Experiences and Adult Household Firearm Ownership. JAMA Netw Open. 2024;7(8):e2428027. doi: 10.1001/jamanetworkopen.2024.28027 39145981 PMC11327881

[39] Centers for Disease Control and Prevention. Behavioral Risk Factor Surveillance System Comparability of Data: BRFSS 2022. Centers for Disease Control and Prevention. 2022.

[40] Liu Y, Yin Z, Ni C, Yan C, Wan Z, Malin B. Examining rural and urban sentiment difference in COVID-19–related topics on Twitter: word embedding–based retrospective study. J Med Internet Res. 2023;25(1):e42985. doi: 10.2196/42985 PMC9937112 36790847

[41] Kaufman BG, Whitaker R, Pink G, Holmes GM. Half of rural residents at high risk of serious illness due to COVID-19, creating stress on rural hospitals. J Rural Health. 2020;36(4):584–90. doi: 10.1111/jrh.12481 32603030 PMC7361543

[42] Peters DJ. Community Susceptibility and resiliency to COVID-19 Across the rural-urban continuum in the United States. J Rural Health. 2020;36(3):446–56. doi: 10.1111/jrh.12477 32543751 PMC7323251

[43] House TW. FACT SHEET: Biden Administration Announces Six-Week Campaign to Get More Americans their Updated COVID-19 Vaccine Before End of the Year. The White House. https://www.whitehouse.gov/briefing-room/statements-releases/2022/11/22/fact-sheet-biden-administration-announces-six-week-campaign-to-get-more-americans-their-updated-covid-19-vaccine-before-end-of-the-year/. 2022. Accessed 2024 August 11.

[44] House TW. FACT SHEET: President Biden to Announce Goal to Administer at Least One Vaccine Shot to 70% of the U.S. Adult Population by July 4th. https://www.whitehouse.gov/briefing-room/statements-releases/2021/05/04/fact-sheet-president-biden-to-announce-goal-to-administer-at-least-one-vaccine-shot-to-70-of-the-u-s-adult-population-by-july-4th/. 2021. Accessed 2024 August 11.

[45] Anda RF, Porter LE, Brown DW. Inside the adverse childhood experience score: strengths, limitations, and misapplications. Am J Prev Med. 2020;59(2):293–5. 32222260 10.1016/j.amepre.2020.01.009

[46] Baldwin JR, Caspi A, Meehan AJ, Ambler A, Arseneault L, Fisher HL, et al. Population vs Individual Prediction of Poor Health From Results of Adverse Childhood Experiences Screening. JAMA Pediatr. 2021;175(4):385–93. doi: 10.1001/jamapediatrics.2020.5602 33492366 PMC7835926

[47] CDC Foundation. Strategies to Improve Communication and Messaging in Rural Populations: Guidance for Supporting Vaccination. CDC Foundation. 2023. https://www.cdcfoundation.org/communications-guidance-rural-populations

[48] Nanyonjo A, Nelson D, Sayers E, Lall P, Vernon-Wilson E, Tetui M, et al. Community efforts to promote vaccine uptake in a rural setting: a qualitative interview study. Health Promot Int. 2023;38(4):daad088. 10.1093/heapro/daad088 PMC10406424 37549195

[49] Christou-Ergos M, Wiley KE, Leask J, Shapiro GK. Traumatic events and vaccination decisions: a systematic review. Vaccines. 2022;10(6):911. 10.3390/vaccines10060911 35746519 PMC9230365

