# Supplementary material for: Adverse childhood experiences and COVID-19 vaccination uptake: Examining the intersection of sex and urban-rural residence
Source: PLoS One. 2025 Nov 7;20(11):e0336390. doi: 10.1371/journal.pone.0336390 (PMC12594379; doi:10.1371/journal.pone.0336390)
Supplement: S1 File — (DOCX) [file pone.0336390.s001.docx]

**Appendix A: Sample Selection Flowchart**

| Participated in 2022 BRFSS | *N* = 445,132  (54 States & Territories) |
| --- | --- |
|  | **↓** |
| Participated in the “Adverse Childhood Experiences” Module | *N* = 47,876  (8 States and Territories) |
|  | **↓** |
| Participated in “COVID Vaccination” | *N* = 15,680  (3 States) |
|  | **↓** |
| Answered Questions on Adverse Childhood Experiences | *N* = 14,472  (3 States) |
|  | **↓** |
| Answered Question on COVID Vaccination | *N* = 14,405  (3 States) |
| Has Data on Control Variables | ↓ |
|  | *N* = 12,085  (3 States) |

**Appendix B: Definitions and Coding of Adverse Childhood Experience Questions**

| Prologue: I'd like to ask you some questions about events that happened during your childhood. This information will allow us to better understand problems that may occur early in life and may help others in the future. This is a sensitive topic and some people may feel uncomfortable with these questions. At the end of this section, I will give you a phone number for an organization that can provide information and referral for these issues. Please keep in mind that you can ask me to skip any question you do not want to answer. All questions refer to the time period before you were 18 years of age. | | | | |
| --- | --- | --- | --- | --- |
| **ACE Item** | **Question** | **Original Coding** | **Coding for Analysis** |  |
| Household Mental Illness | Did you live with anyone who was depressed, mentally ill, or suicidal? | 1 Yes  2 No  7 Don't Know/Not Sure  9 Refused | 0 No  1 Yes  *Don't Know/Not Sure or Refused recoded as missing |  |
| Household Alcoholism | Did you live with anyone who was a problem drinker or alcoholic? | 1 Yes  2 No  7 Don't Know/Not Sure  9 Refused | 0 No  1 Yes  *Don't Know/Not Sure or Refused recoded as missing |  |
| Household Illegal Drug Use | Did you live with anyone who used illegal street drugs or who abused prescription medications? | 1 Yes  2 No  7 Don't Know/Not Sure  9 Refused | 0 No  1 Yes  *Don't Know/Not Sure or Refused recoded as missing |  |
| Household Incarceration | Did you live with anyone who served time or was sentenced to serve time in a prison, jail, or other correctional facility? | 1 Yes  2 No  7 Don't Know/Not Sure  9 Refused | 0 No  1 Yes  *Don't Know/Not Sure or Refused recoded as missing |  |
| Parents Divorced or Separated | Were your parents separated or divorced? | 1 Yes  2 No  7 Don't Know/Not Sure  8 Parents not married  9 Refused | 0 No or not Married  1 Yes  *Don't Know/Not Sure or Refused recoded as missing |  |
| Household Domestic Violence | How often did your parents or adults in your home ever slap, hit, kick, punch or beat each other up? Was it… | 1 Never  2 Once  3 More than once  7 Don't Know/Not Sure  9 Refused | 0 Never  1 Once or more  *Don't Know/Not Sure or Refused recoded as missing |  |
| Physical Abuse | Not including spanking, (before age 18), how often did a parent or adult in your home ever hit, beat, kick, or physically hurt you in any way? Was it… | 1 Never  2 Once  3 More than once  7 Don't Know/Not Sure  9 Refused | 0 Never  1 Once or more  *Don't Know/Not Sure or Refused recoded as missing |  |
| Verbal Abuse | How often did a parent or adult in your home ever swear at you, insult you, or put you down? Was it… | 1 Never  2 Once  3 More than once  7 Don't Know/Not Sure  9 Refused | 0 Never  1 Once or more  *Don't Know/Not Sure or Refused recoded as missing |  |
| Sexual Abuse | (1) How often did anyone at least 5 years older than you or an adult, ever touch you sexually? Was it…  (2) How often did anyone at least 5 years older than you or an adult, try to make you touch them sexually? Was it…  (3) How often did anyone at least 5 years older than you or an adult, force you to have sex? Was it… | 1 Never  2 Once  3 More than once  7 Don't Know/Not Sure  9 Refused | 0 Never to all 3 questions  1 Once or more to any of the 3 questions  *Don't Know/Not Sure or Refused recoded as missing |  |

**Appendix C: Definitions and Coding of COVID-19 Vaccination, Moderating Variables, and Control Variables**

| **Variable** | **Question** | **Original Coding** | **Coding for Analysis** |
| --- | --- | --- | --- |
| COVID-19 Vaccination | Have you received at least one dose of a COVID-19 vaccination? | 1 Yes  2 No  7 Don't Know/Not Sure  9 Refused | 0 No  1 Yes  *Don't Know/Not Sure or Refused recoded as missing |
| Sex | Are you male or female? | 1 Male  2 Female | 0 Female  1 Male |
| Urbanicity | Urban/Rural Status | 1 Urban counties  2 Rural counties | 0 Urban counties  1 Rural counties |
| Age | Calculated variable for six-level age category (_age_g) | 1 age 18 to 24  2 age 25 to 34  3 age 35 to 44  4 age 45 to 54  5 age 55 to 65  6 age 65 or older | 1 age 18 to 24  2 age 25 to 34  3 age 35 to 44  4 age 45 to 54  5 age 55 to 65  6 age 65 or older |
| Race/Ethnicity | (1) Which one or more of the following would you say is your race?  (2) Are you Hispanic, Latino/a, or Spanish origin? | **(1) Race**  10 White  20 Black or African American  30 American Indian or Alaska Native  40 Asian  41 Asian Indian  42 Chinese  43 Filipino  44 Japanese  45 Korean  46 Vietnamese  47 Other Asian  50 Pacific Islander  51 Native Hawaiian  52 Guamanian or Chamorro  53 Samoan  54 Other Pacific Islander  88 No choices  77 Don't know / Not sure  99 Refused  **(2) Hispanic/Latino/a Ethnicity**  Are you…  1 Mexican, Mexican American, Chicano/a  2 Puerto Rican  3 Cuban  4 Another Hispanic, Latino/a, or Spanish origin  5 No  7 Don't know / Not sure  9 Refused | 0 non-Hispanic White  1 non-Hispanic Black  2 Hispanic  3 non-Hispanic other Race  *Don't Know/Not Sure or Refused recoded as missing |
| Marital Status | Are you… | 1 Married  2 Divorced  3 Widowed  4 Separated  5 Never married  6 A member of an unmarried couple  9 Refused | 1 Married  2 Divorced or Seperated  3 Widowed  4 Never married  5 A member of an umarried couple  *Refused recoded as missing |
| Educational Attainment | What is the highest grade or year of school you completed? | 1 Never attended school or only attended kindergarten  2 Grades 1 through 8 (Elementary)  3 Grades 9 through 11 (Some high school)  4 Grade 12 or GED (High school graduate)  5 College 1 year to 3 years (Some college or technical school)  6 College 4 years or more (College graduate)  9 Refused | 0 Less than high school  1 High school graduate  2 Some college  3 College graduate  *Refused recoded as missing |
| Child in Home | How many children less than 18 years of age live in your household? | -- Number of children  88 None  99 Refused | 0 No children  1 One or more Children  *Don't Know/Not Sure or Refused recoded as missing |
| Military Veteran | Have you ever served on active duty in the United States Armed Forces, either in the regular military or in a National Guard or military reserve unit? | 1 Yes  2 No  7 Don't know / Not sure  9 Refused | 0 No  1 Yes  *Don't Know/Not Sure or Refused recoded as missing |
| Household Income | Is your annual household income from all sources: | 1 Less than $10,000?  2 Less than $15,000? ($10,000 to less than $15,000)  3 Less than $20,000? ($15,000 to less than $20,000)  4 Less than $25,000  5 Less than $35,000 If ($25,000 to less than $35,000)  6 Less than $50,000 If ($35,000 to less than $50,000)  7 Less than $75,000? ($50,000 to less than $75,000)  8 Less than $100,000? ($75,000 to less than $100,000)  9 Less than $150,000? ($100,000 to less than $150,000)?  10 Less than $200,000? ($150,000 to less than $200,000)  11 $200,000 or more  77 Don't know / Not sure  99 Refused | 1 Less than $25,000  2 $25,000 - $49,999  3 $50,000 - $74,999  4 $75,000 - $99,999  5 $100,000 - $149,000  6 $150,000 or more  *Don't Know/Not Sure or Refused recoded as missing |
| Survey Language | Language Identifier | 1 English  2 Spanish | 0 Spanish  1 English |

**Appendix D: Multivariable Logistic Regression of COVID-19 Vaccination on Type of ACE**

|  | **Full Sample**  **(N = 12,085)** | **Urban Female**  **(N = 4,368)** | **Urban Male**  **(N = 4,297)** | **Rural Female**  **(N = 1,718)** | **Rural Male**  **(N = 1,702)** |
| --- | --- | --- | --- | --- | --- |
| **Type of ACE^a,b^** | **aOR (95% CI)** | **aOR (95% CI)** | **aOR (95% CI)** | **aOR (95% CI)** | **aOR (95% CI)** |
| Household Mental Illness | 1.12 (0.95-1.32) | 1.19 (0.91-1.57) | 1.28 (0.97-1.70) | 1.02 (0.68-1.51) | 0.56* (0.36-0.88) |
| Household Alcoholism | 0.99 (0.85-1.15) | 0.90 (0.70-1.16) | 1.10 (0.86-1.42) | 1.15 (0.78-1.69) | 0.78 (0.52-1.16) |
| Household Illegal Drug Use | 0.82* (0.68-1.00) | 0.94 (0.68-1.31) | 0.74 (0.55-1.01) | 0.86 (0.53-1.41) | 0.72 (0.38-1.37) |
| Household Incarceration | 0.74** (0.59-0.92) | 0.66** (0.45-0.97) | 0.74 (0.52-1.06) | 0.74 (0.41-1.35) | 1.11 (0.58-2.14) |
| Parents Divorced or Separated | 0.90 (0.78-1.04) | 0.87 (0.68-1.13) | 0.99 (0.78-1.26) | 1.02 (0.69-1.52) | 0.75 (0.52-1.07) |
| Household Domestic Violence | 0.94 (0.79-1.12) | 0.95 (0.71-1.27) | 0.96 (0.72-1.28) | 1.02 (0.66-1.57) | 0.74 (0.46-1.18) |
| Physical Abuse | 0.94 (0.81-1.10) | 0.88 (0.68-1.15) | 0.96 (0.75-1.23) | 1.31 (0.89-1.94) | 0.75 (0.52-1.08) |
| Verbal Abuse | 1.00 (0.87-1.14) | 0.93 (0.73-1.19) | 1.03 (0.82-1.28) | 1.15 (0.82-1.62) | 0.90 (0.65-1.24) |
| Sexual Abuse | 0.70*** (0.58-0.85) | 0.66** (0.50-0.88) | 0.86 (0.58-1.28) | 0.76 (0.52-1.12) | 0.55 (0.26-1.16) |

*** p<0.001, ** p<0.01, * p<0.05

*Abbreviations*: ACEs = adverse childhood experiences; aOR = adjusted odds ratio; CI = confidence interval

^a^ Control variables include age, race/ethnicity, marital status, educational attainment, child in the home, veteran status, household income, and survey language. In the regression model of the full sample, sex and urbanicity act as additional control variables.

^b^ Each row in represents a separate multiple logistic regression analysis.
